# DDR-mediated crosstalk between DNA-damaged cells and their microenvironment

**DOI:** 10.3389/fgene.2015.00094

**Published:** 2015-03-12

**Authors:** Nicolas Malaquin, Audrey Carrier-Leclerc, Mireille Dessureault, Francis Rodier

**Affiliations:** ^1^Centre de Recherche du Centre Hospitalier de l’Universite de Montréal (CRCHUM), et Institut du cancer de Montréal,Montreal, QC, Canada; ^2^Département de Radiologie, Radio-Oncologie et Médicine Nucléaire, Université de MontréalMontreal, QC, Canada

**Keywords:** DNA damage response, senescence, bystander effect, senescence secretome, inflammation, microenvironment, tissue damage

## Abstract

The DNA damage response (DDR) is an evolutionarily conserved signaling cascade that senses and responds to double-strand DNA breaks by organizing downstream cellular events, ranging from appropriate DNA repair to cell cycle checkpoints. In higher organisms, the DDR prevents neoplastic transformation by directly protecting the information contained in the genome and by regulating cell fate decisions, like apoptosis and senescence, to ensure the removal of severely damaged cells. In addition to these well-studied cell-autonomous effects, emerging evidence now shows that the DDR signaling cascade can also function in a paracrine manner, thus influencing the biology of the surrounding cellular microenvironment. In this context, the DDR plays an emerging role in shaping the damaged tumor microenvironment through the regulation of tissue repair and local immune responses, thereby providing a promising avenue for novel therapeutic interventions. Additionally, while DDR-mediated extracellular signals can convey information to surrounding, undamaged cells, they can also feedback onto DNA-damaged cells to reinforce selected signaling pathways. Overall, these extracellular DDR signals can be subdivided into two time-specific waves: a rapid bystander effect occurring within a few hours of DNA damage; and a late, delayed, senescence-associated secretory phenotype generally requiring multiple days to establish. Here, we highlight and discuss examples of rapid and late DDR–mediated extracellular alarm signals.

The DNA damage response (DDR) signaling network is essential in the maintenance of genomic stability, via the initiation and coordination of DNA repair mechanisms with appropriate cell cycle arrest checkpoints (d’Adda di Fagagna, 2008; Jackson and Bartek, 2009). The DDR is initially propagated by a series of effective and rapid post-translational modifications culminating in the activation of nodal transcription factors like p53, which organize additional DDR transcriptional responses (Harper and Elledge, 2007).

Briefly, a typical DDR cascade begins with the recruitment and activation of an apical DDR kinase like ATM (ataxia-telangiectasia mutated) to DNA double-strand breaks (DSBs) by damage sensors such as the MRN complex (MRE-11, Rad-51 and NBS-1 proteins). This leads to the local phosphorylation of multiple ATM substrates in the chromatin surrounding the DNA lesion, almost always including the histone variant H2AX (phospho-H2AX or üH2AX). These local chromatin modifications provoke the further recruitment of additional DDR mediators at the break, including 53BP1 and MDC1, which amplify chromatin modifications over megabases of DNA generating macroscopic structures called DNA damage foci (DDF; Rogakou et al., 1998; Bonner et al., 2008) that allows for the direct visualization of single DSBs in mammalian cell nuclei (Rogakou et al., 1999). Simultaneously, the distal propagation of the DDR signal within the cell promote cell cycle checkpoints and the activation of p53 (Rodier et al., 2007). When DNA lesions are repairable, the ensuing growth arrest is transient, eventually resulting in cell cycle resumption, and a return to normality. In contrast, severe or irreparable DNA lesions trigger prolonged DDR signaling, resulting in apoptosis or senescence (permanent growth arrest; Campisi and d’Adda di Fagagna, 2007).

## The DDR Generates Extracellular Signals

**FIGURE 1:**
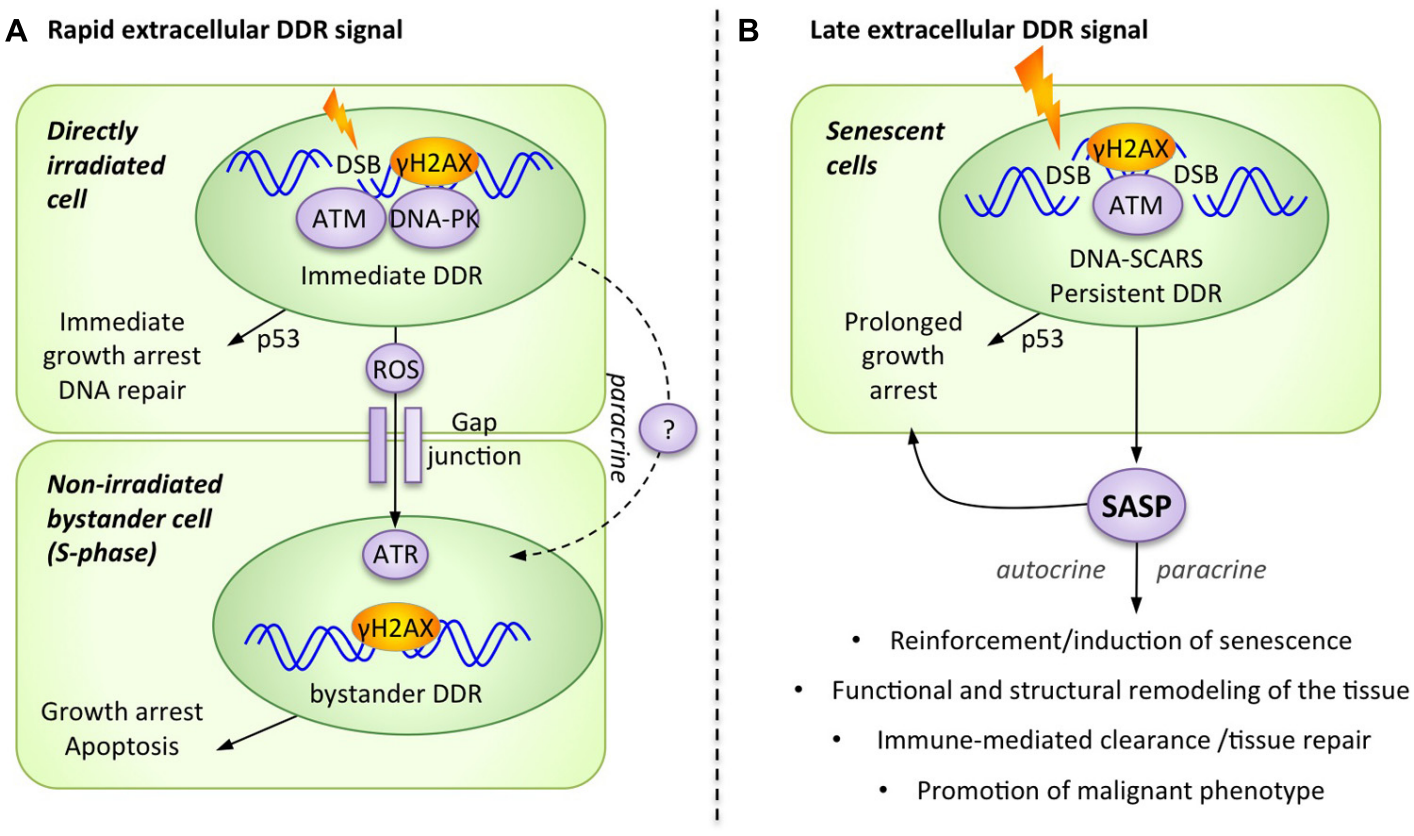
**The DNA damage response (DDR) generates alarm signals that are transmitted from the DNA-damaged cell to the extracellular microenvironment. (A)** Rapid extracellular DDR signals occur in response to DNA damage and are transmitted to neighboring cells via direct cell–cell contact and paracrine signals. **(B)** Late extracellular DDR signals occur in response to persistent DNA damage signaling and are collectively known as the senescence-associated secretory phenotype (SASP).

The DDR is mostly known for its role as a cell-autonomous, intracellular signaling cascade that regulates DNA repair and cell cycle checkpoints. However, in the context of higher organisms with multicellular tissues, cells have developed intricate intercellular communication mechanisms that the DDR employs to trigger extracellular alarm signals. Conceptually, it is entirely plausible that damaged cells can signal to other cells that their genome has been compromised, essentially generating tissue-wide stress responses. In fact, these DDR-mediated extracellular alarm signals can be subdivided into at least two waves: rapid and late. While we are still far from a complete understanding of extracellular DDR signaling, it is already well established that specific communication mechanisms including cell surface bound and soluble molecules are involved in this process (**Figure 1**). Bystander responses received by cells adjacent to damaged cells have been described, and more importantly, some soluble signals have been proposed to travel further in the body, creating additional potential therapeutic intervention opportunities (Tchkonia et al., 2013; Havaki et al., 2014).

### A Rapid Extracellular DDR Signal Reaches Undamaged Bystander Cells

Accumulating experimental evidence shows that damaged cells rapidly transmit a DDR-dependent stress signal to neighboring healthy cells, provoking paracrine activation of stress responses such as a bystander DDR. While not originally linked to the DDR itself, this phenomenon was first described under conditions in which only 1% of the cells in a population were irradiated by a low dose of alpha-particles, yet 30% of the cells exhibited chromosomal changes (Nagasawa and Little, 1992). This bystander damage response could be an important mechanism used to rapidly amplify the effect of low dose irradiation by transferring DNA-damage signals from irradiated cells to non-irradiated ones.

It is now clear that non-irradiated cells can adopt common DNA damage-associated phenotypes from adjacent irradiated cells, including micronuclei formation, altered expression of stress-related genes, various epigenetic changes, increased frequency of mutations, induction of apoptosis or senescence, and even malignant transformation (Azzam et al., 2002; Nagasawa and Little, 2002; Morgan, 2003; Ko et al., 2006). Interesting mechanistic evidence supporting the activation of the DDR in bystander cells includes the formation of DNA damage foci (DDF), which also suggests the accumulation of DSBs in these cells (**Figure 1A**; Sokolov et al., 2007). The formation of bystander γH2AX foci has been observed in a number of experimental systems, including human cultured cells and three-dimensional tissue models, as well as *in vivo* mouse models (Sokolov et al., 2005; Sedelnikova et al., 2007). Furthermore, normal fibroblasts that were exposed to damaged cells, either directly through co-culture or indirectly through conditioned media, demonstrated many other typical DDR markers in DDF, including 53BP1, phospho-ATM, and the focal presence of the ATM-activating MRN complex (Sokolov et al., 2005; Sedelnikova et al., 2007).

The pathways involved in the transmission of alarm signals generated by irradiated cells remain ill defined, but emerging insight appears promising. For example, the activation of DNA-PKcs and ATM is necessary for the generation of a bystander signal from the damaged cell, but these kinases are not required for signal reception in non-irradiated bystander cells (Hagelstrom et al., 2008). Alternatively, the kinase ATR could be required in the recipient bystander cell to allow for the formation of DDF (containing γH2AX, 53BP1, BRCA1) and the subsequent activation of ATM. Importantly, this ATR-dependent bystander DDR activation occurs only in S-phase cells, consistent with the concept that replication stress is a major trigger for ATR activation (Burdak-Rothkamm et al., 2007, 2008). Accordingly, the radiation-triggered extracellular alarm signal preferentially affects non-irradiated cells that display high rates of replication and transcriptional activities (Dickey et al., 2012). Overall, this suggests that not all bystander cells equally trigger a bystander DDR, and that actively dividing cells are most receptive to this signal (**Figure 1A**).

Two distinct pathways for the transmission of rapid extracellular DDR signals have been proposed: direct cell–cell communication and paracrine interaction (**Figure 1A**). For cells in direct physical contact, small molecules (<1.5 kDa) are usually transmitted through multimeric protein channels termed gap junctions, and the rapid extracellular DDR signal is effectively abrogated following the use of pharmacological inhibitors against gap junctions (i.e., lindane) or by the genetic ablation of an essential gap junction component, connexin 43 (Azzam et al., 1998, 2001). To directly communicate with neighboring cells, the DDR has also been shown to increase the presence of selected cell surface ligands and receptors on damaged cells. For example, some DDR regulated cell surface molecules can subsequently engage surrounding immune cells (NKG2D ligands) or can influence damaged cells survival (DR5 receptor) via receptor-ligand engagement (Wu et al., 1997; Finnberg et al., 2005; Gasser et al., 2005; Lam et al., 2014). A second signaling route consists of the release of soluble factors into the extracellular media, which act in a paracrine manner to stimulate neighboring cells. Consistent with this mechanism, the addition of conditioned media from irradiated cells is sufficient to induce DDF and bystander DDR activation in non-irradiated cells (Sokolov et al., 2005; Shao et al., 2008; Dickey et al., 2009; Klammer et al., 2010).

The molecular players directly tasked with conveying rapid stress signaling from cell to cell are still poorly defined. The most commonly described family of factors is reactive oxygen or nitrogen species (ROS/NOS), produced at high levels in the damaged cell (Havaki et al., 2014). Indeed, the activation of the DDR as well as its downstream phenotypes in bystander cells (i.e., up-regulation of stress genes, micronucleus formation) is suppressed by superoxide dismutase activation or by ROS inhibitors (Azzam et al., 2002; Little et al., 2002). ROS, and in particular H_2_O_2,_ which has a relatively longer half-life, can freely diffuse across plasma membranes or through gap junctions, causing DNA damage at distant sites (Azzam et al., 2003). Oxidative stress can result in DNA lesions in the form of single strand DNA breaks (SSBs) that can be converted to DSBs when unresolved or abundant, suggesting that ROS can account for at least a subset of the observed bystander DNA damage events (Tanaka et al., 2006). The second class of soluble factors involved in long distance extracellular DDR signaling includes molecules such as transforming growth factor-β1 (TGF-β1) and tumor necrosis factor-α (TNF-α; Iyer et al., 2000). In addition to its direct role in signaling, the TGF-β1 secreted by the irradiated cells also contributes to the intracellular increase of ROS and NOS in bystander cells, most likely through NAD(P)H oxidase activation (Burdak-Rothkamm et al., 2007, 2008; Shao et al., 2008). Some, and perhaps most, rapid intercellular damage signaling processes also play a role in the late extracellular response (see below). However, the opposite is not necessarily true, for example, cytokines like IL-6 and IL-8 are exclusive to the late phase following irradiation (Rodier et al., 2009).

### A Late Senescence-Associated Extracellular DDR Signal Modifies the Microenvironment

In general, the early phase of the intracellular DDR signaling cascade is a well-established response to nuclear damage, occurring within seconds to hours of the initial assault. But when DNA lesions are particularly severe or irreparable, such as uncapped telomeres (d’Adda di Fagagna et al., 2003), the DDR signal can persist and provoke programmed cell death (apoptosis) or permanent growth arrest (cell senescence; Rodier and Campisi, 2011). While apoptotic cells are rapidly eliminated, damaged senescent cells can persist for extended periods and accumulate in damaged or aging tissues (Baker et al., 2011). Senescence typically depends on the p53/p21 and p16INK4a/RB tumor suppressor pathways (Campisi, 2003; d’Adda di Fagagna, 2008) and is characterized by a series of functional hallmarks (Rodier and Campisi, 2011; Lopez-Otin et al., 2013). It is important to note that the DDR remains permanently activated in most senescent cells, as evidenced by the presence of persistent DDF, termed “DNA segment with chromatin alterations reinforcing senescence” (DNA-SCARS; Rodier et al., 2011). These DNA-SCARS, whether telomeric or intra-chromosomal, are suggested DDR activity nodes that maintain long-term DDR signaling (Rodier et al., 2011).

With few exceptions (Coppe et al., 2011), senescent cells from most species and tissues that are triggered by various stresses all display a Senescence-Associated Secretory Phenotype (SASP; **Figure 1B**), which is critical for the ability of these cells to modulate their microenvironment (Coppe et al., 2008, 2010a,b; Ohanna et al., 2011). A large subset of this SASP critically depends on DDR signaling and is thus an extracellular extension of the DDR (Rodier et al., 2009). The SASP is defined as a pro-inflammatory secretome composed of cytokines (i.e., IL-6 IL-8, GROα, GROβ, MCP-1), growth factors (i.e., GM-CSF, G-CSF, HGF/SF, IGF), proteases (i.e., metalloproteinase MMP-1, -2, and -3), and other non-soluble extracellular matrix proteins (i.e., collagens, fibronectin, laminin; Bavik et al., 2006; Coppe et al., 2008, 2010a; Ohanna et al., 2011; Malaquin et al., 2013). The exact composition of the SASP, its targets, and the overall downstream outcomes vary considerably depending on the cellular context and the type of stresses, but the consensus is that the SASP is at least partially DDR-dependent and is in major part responsible for modulating senescence-associated inflammatory microenvironments in tissues (**Figure 1B**).

The SASP contributes to senescence reinforcement in damaged cells and to tissue repair, but also to age-associated tissue dysfunction and other age-related diseases, including cancer (**Figure 1B**). Because the SASP appears to have both beneficial and deleterious effects, it may represent an interesting, double-edged target for pharmaceutical intervention in human disease (Acosta and Gil, 2012; Perez-Mancera et al., 2014). In the context of cancer, which is particularly applicable to DDR events activated by irradiation or chemotherapy, the SASP also contributes to the clearance of damaged senescent tumor cells by enhancing both innate and adaptive immunity (Xue et al., 2007; Kang et al., 2011; Iannello et al., 2013). However, the SASP also generates chronic inflammation in normal tissues with persistent senescent cells, contributing to age-related tissue dysfunction (Rodier and Campisi, 2011). In the case of the tumor microenvironment, the SASP of senescent stromal fibroblasts sustains tumor growth and invasion and can even create tumor microenvironments that promote long-term cancer therapy resistance (Krtolica et al., 2001; Sun et al., 2012). Overall, understanding the molecular regulation of the SASP appears essential to reveal how the DDR manages extracellular signaling.

## Molecular Regulation of the SASP by the DDR

**FIGURE 2:**
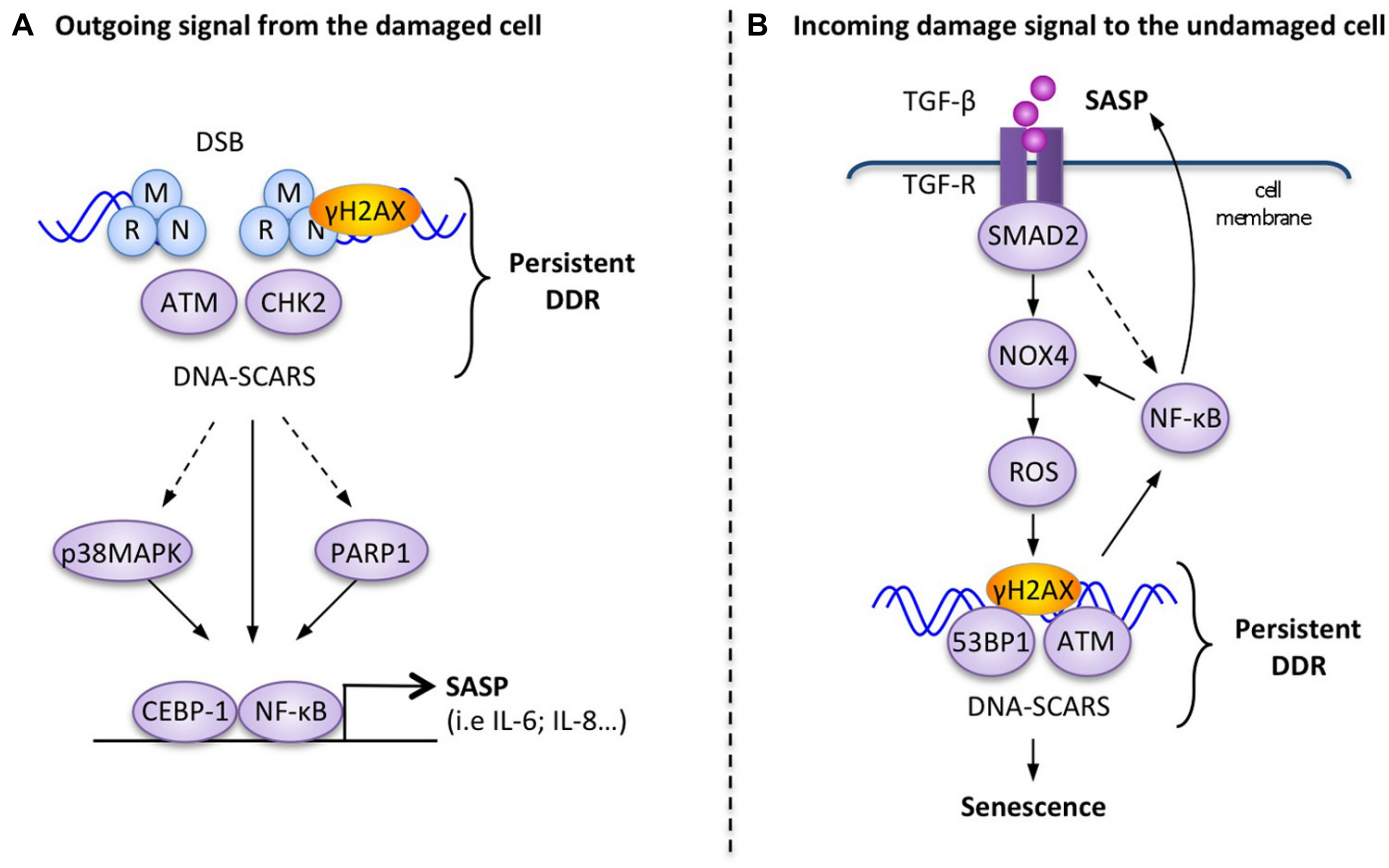
**Examples of molecular interactions between the DDR and outgoing–incoming extracellular damage signals. (A)** Outgoing signal from the damaged cell: in response to persistent DNA-SCARS, molecular components of the DDR cascade lead to selected transcription factor activation and increased transcription of SASP factors such as IL-6. **(B)** Incoming damage to the undamaged cell: the presence of extracellular TGF-β can reinforce DDR-mediated p53 activity and trigger the formation of DNA-SCARS, which subsequently mediate senescence phenotypes, including increased secretion of SASP factors that reinforce a positive senescence feedback loop.

Direct molecular links between the SASP and the DDR have been demonstrated (**Figure 2A**), but unlike the rapid extracellular DDR signals, the SASP is a slow, delayed response to DDR signaling. While apical DDR kinases like ATM are activated within minutes of DNA lesions and subsequent DDR transcriptional responses are established within hours by p53 and other transcription factors, the SASP develops over days, with associated factors like IL-6 reaching maximal secretion levels 4–10 days after DDR initiation (Coppe et al., 2008; Rodier et al., 2009). In response to DNA damage, persistent DDR signals emanating from DNA-SCARS are necessary, both for the establishment and maintenance of the SASP (Rodier et al., 2009, 2011). At the molecular level, the DDR proteins H2AX, ATM, NBS1 and CHK2, but not cell cycle arrest mediators p53 and pRb, are required to support the SASP (Rodier et al., 2009, 2011). Activation of the p38MAPK stress kinase pathway also triggers the SASP and in some situations concurrent activation of the DDR is not necessary suggesting that there may be different subsets of SASP factors requiring varying levels of interaction with the DDR (**Figure 2A**; Freund et al., 2011). For example, the depletion of ATM completely prevents the secretion of IL-6 and IL-8 in senescent irradiated human fibroblasts, but does not impede increased secretion of MCP1, TIMP2, and IGFBP2 (Rodier et al., 2009).

The inflammation-associated transcription factor, nuclear factor-kB (NF-κB), is revealing itself to be a master regulator of the SASP (**Figure 2A**; Salminen et al., 2012). The activation of the RelA p65 subunit of NF-κB and its recruitment to the chromatin are necessary for the expression of several SASP factors, including IL-6 and IL-8 (Chien et al., 2011). Several studies also showed that the DDR can directly trigger activation of NF-κB signaling via the interaction between activated ATM and the NEMO protein, which is a regulatory subunit of the IKK complex (inhibitor of NF-κB signaling). DDR activation results in the export of an ATM/NEMO complex into the cytoplasm, where it binds to and activates IKKα/β, leading to the initiation of NF-κB signaling via the phosphorylation of inhibitory IκB proteins (Huang et al., 2003; Wu et al., 2006; Miyamoto, 2011). C/EBPβ (CCAAT-enhancer-binding proteins), another transcription factor known to be involved in inflammatory regulation, can also contribute to SASP induction in cooperation with NF-κB (Kuilman et al., 2008).

Alternatively, another important DNA-damage sensor and DDR regulator, known as PARP-1 (Poly-ADP-ribose polymerase 1), is also involved in the regulation of NF-kB in senescent melanoma cells undergoing the SASP (Ohanna et al., 2011). Perhaps linked in this context, activated PARP-1 can interact with NEMO to enhance the formation of the ATM/NEMO complex (Stilmann et al., 2009).

## Cell-Autonomous Reinforcement or Bystander Activation of the DDR Using Late Extracellular DDR Signals (SASP)

Much like the bystander effect described for rapid DDR extracellular signals, the SASP generated from persistently damaged cells is known to modulate DDR-associated behaviors in neighboring cells. Although ROS may influence how the DDR generates the SASP (Guo et al., 2010), most of the SASP’s known extracellular effects are currently associated with proteinic soluble factors. Additionally, and again in contrast to the rapid DDR extracellular response, the SASP has been shown to impact both the signal-emitting damaged cell and healthy bystander cells. In damaged cells, the SASP can reinforce p53-associated DDR pathways in a paracrine manner, which maintains senescence in these cells. For example, IL-6 is considered to be a major mediator of paracrine senescence reinforcement (Kuilman et al., 2008). Similarly, CXCR2-binding chemokines (such as IL-8 or GRO-1) are also crucial to reinforce oncogenic- and replication-induced senescence (Acosta et al., 2008). Alternatively, the SASP generated by senescent cells also impacts neighboring bystander cells, as demonstrated both in culture and *in vivo* (Kuilman et al., 2008; Nelson et al., 2012; Acosta et al., 2013). In particular, multiple SASP components secreted by oncogene-induced senescent cells can trigger paracrine senescence in bystander cells (i.e., TGFβ family ligands, VEGF, CCL2, and CCL20) and IL-1 signaling is apparently a major upstream regulator of this paracrine senescence (Acosta et al., 2013). Finally, the SASP factor MCP-1 (CCL2), found in the conditioned media of senescent melanoma cells, was demonstrated to promote DNA lesions in other cells, as illustrated by an increase in 53BP1 DDF (Ohanna et al., 2011). Other extracellular signals that are not necessarily secreted by damaged or senescent cells can also connect to the DDR. For example, type I β-interferon secreted by virally infected cells has been shown to induce paracrine bystander senescence in other cells via the generation of ROS, DDR activation, and p53 activity (Moiseeva et al., 2006).

The link between extracellular signaling and DDR activation is well illustrated by TGFβ signaling, which is often associated with senescence (Hubackova et al., 2012; **Figure 2B**). The inhibition of the TGFβ pathway resulted in defective DDR activation in irradiated normal cells, as measured by decreased p53 activation and a reduction in ATM, CHK2, and H2AX phosphorylation (Kirshner et al., 2006). The addition of recombinant TGFβ-1 also restored functional ATM in damaged normal cells and could induce DDR-associated senescent phenotypes in healthy hepatocellular carcinoma cells (Kirshner et al., 2006; Senturk et al., 2010). Similarly, TGFβ-1 from the conditioned media of senescent normal fibroblasts (oncogene-induced senescent, replicative exhaustion, or genotoxic drugs) triggered a senescent growth arrest in undamaged cells via the DDR-associated p53 or the p16 pathways (**Figure 2B**). TGFβ-induced bystander senescence is associated with the activation of a persistent DDR, the formation of DNA-SCARS, and the subsequent production of SASP factors. It is probable that the activation of the TGFβ/SMAD pathway results in increased intracellular ROS and NOS production in the target bystander cells through an NF-κB-mediated increase in Nox4 expression and NAPDH oxidase activity (Burdak-Rothkamm et al., 2007, 2008; Shao et al., 2008). Finally, the stimulation of the IL1R/NF-κB pathway known to activate cellular inflammatory responses also cooperates with TGFβ/SMAD to induce bystander senescence (Hubackova et al., 2012).

## Conclusion and Perspectives

It is now clear that DNA-damaged cells interact with the extracellular environment to induce bi-directional changes within themselves and in undamaged neighboring cells. These communication strategies have most likely evolved to convey stress signals from damaged cells to the surrounding tissue and occur relatively rapidly (within hours) and/or slowly under the shape of the SASP. In the case of cancer treatment, therapeutic tools, including radiation and cytotoxic drugs, can trigger DDR activity and cellular senescence in normal and neoplastic cells but whether the generation of a DDR-driven immunomodulatory microenvironment has beneficial or detrimental consequences remains unknown (Acosta and Gil, 2012; Sun and Nelson, 2012). It is thus evident that understanding microenvironment-modulating DDR-related mechanisms and their consequences remains a major challenge in the development of successful cancer therapies. Recent tools have emerged to directly manipulate senescence in mammalian model systems, which will be very useful in determining the importance of extracellular signals emitted from senescent cells (Baker et al., 2011; Laberge et al., 2013; Demaria et al., 2014). The use of these models and other strategies will be instrumental in the exploration of the pathways regulating DDR-mediated extracellular communication, as well as in the identification of extracellular signaling molecules that may become potential targets for therapeutic development in advanced cancer therapies that take into account tissue microenvironments.

## References

[B1] Acosta J. C. Banito A. Wuestefeld T. Georgilis A. Janich P. Morton J. P. (2013). A complex secretory program orchestrated by the inflammasome controls paracrine senescence. *Nat. Cell Biol.* 15 978–990 10.1038/ncb2784 23770676 PMC3732483

[B2] Acosta J. C. Gil J. (2012). Senescence: a new weapon for cancer therapy. *Trends Cell Biol.* 22 211–219 10.1016/j.tcb.2011.11.006 22245068

[B3] Acosta J. C. O’loghlen A. Banito A. Guijarro M. V. Augert A. Raguz S. (2008). Chemokine signaling via the CXCR2 receptor reinforces senescence. *Cell* 133 1006–1018 10.1016/j.cell.2008.03.038 18555777

[B4] Azzam E. I. De Toledo S. M. Gooding T. Little J. B. (1998). Intercellular communication is involved in the bystander regulation of gene expression in human cells exposed to very low fluences of alpha particles. *Radiat. Res.* 150 497–504 10.2307/3579865 9806590

[B5] Azzam E. I. De Toledo S. M. Little J. B. (2001). Direct evidence for the participation of gap junction-mediated intercellular communication in the transmission of damage signals from alpha- particle irradiated to nonirradiated cells. *Proc. Natl. Acad. Sci. U.S.A.* 98 473–478 10.1073/pnas.011417098 11149936 PMC14611

[B6] Azzam E. I. De Toledo S. M. Little J. B. (2003). Oxidative metabolism, gap junctions and the ionizing radiation-induced bystander effect. *Oncogene* 22 7050–7057 10.1038/sj.onc.1206961 14557810

[B7] Azzam E. I. De Toledo S. M. Spitz D. R. Little J. B. (2002). Oxidative metabolism modulates signal transduction and micronucleus formation in bystander cells from alpha-particle-irradiated normal human fibroblast cultures. *Cancer Res.* 62 5436–5442. 12359750

[B8] Baker D. J. Wijshake T. Tchkonia T. Lebrasseur N. K. Childs B. G. Van De Sluis B. (2011). Clearance of p16Ink4a-positive senescent cells delays ageing-associated disorders. *Nature* 479 232–236 10.1038/nature10600 22048312 PMC3468323

[B9] Bavik C. Coleman I. Dean J. P. Knudsen B. Plymate S. Nelson P. S. (2006). The gene expression program of prostate fibroblast senescence modulates neoplastic epithelial cell proliferation through paracrine mechanisms. *Cancer Res.* 66 794–802 10.1158/0008-5472.CAN-05-1716 16424011

[B10] Bonner W. M. Redon C. E. Dickey J. S. Nakamura A. J. Sedelnikova O. A. Solier S. (2008). GammaH2AX and cancer. *Nat. Rev. Cancer* 8 957–967 10.1038/nrc2523 19005492 PMC3094856

[B11] Burdak-Rothkamm S. Rothkamm K. Prise K. M. (2008). ATM acts downstream of ATR in the DNA damage response signaling of bystander cells. *Cancer Res.* 68 7059–7065 10.1158/0008-5472.CAN-08-0545 18757420 PMC2528059

[B12] Burdak-Rothkamm S. Short S. C. Folkard M. Rothkamm K. Prise K. M. (2007). ATR-dependent radiation-induced gamma H2AX foci in bystander primary human astrocytes and glioma cells. *Oncogene* 26 993–1002 10.1038/sj.onc.1209863 16909103

[B13] Campisi J. (2003). Cancer and ageing: rival demons? *Nat. Rev. Cancer* 3 339–349 10.1038/nrc1073 12724732

[B14] Campisi J. d’Adda di Fagagna F. (2007). Cellular senescence: when bad things happen to good cells. *Nat. Rev. Mol. Cell Biol.* 8 729–740 10.1038/nrm2233 17667954

[B15] Chien Y. Scuoppo C. Wang X. Fang X. Balgley B. Bolden J. E. (2011). Control of the senescence-associated secretory phenotype by NF-kappaB promotes senescence and enhances chemosensitivity. *Genes Dev.* 25 2125–2136 10.1101/gad.17276711 21979375 PMC3205583

[B16] Coppe J. P. Desprez P. Y. Krtolica A. Campisi J. (2010a). The senescence-associated secretory phenotype: the dark side of tumor suppression. *Annu. Rev. Pathol.* 5 99–118 10.1146/annurev-pathol-121808-102144 20078217 PMC4166495

[B17] Coppe J. P. Patil C. K. Rodier F. Krtolica A. Beausejour C. M. Parrinello S. (2010b). A human-like senescence-associated secretory phenotype is conserved in mouse cells dependent on physiological oxygen. *PLoS ONE* 5:e9188 10.1371/journal.pone.0009188 PMC2820538 20169192

[B18] Coppe J. P. Patil C. K. Rodier F. Sun Y. Munoz D. P. Goldstein J. (2008). Senescence-associated secretory phenotypes reveal cell-nonautonomous functions of oncogenic RAS and the p53 tumor suppressor. *PLoS Biol.* 6:2853–2868 10.1371/journal.pbio.0060301 19053174 PMC2592359

[B19] Coppe J. P. Rodier F. Patil C. K. Freund A. Desprez P. Y. Campisi J. (2011). Tumor suppressor and aging biomarker p16(INK4a) induces cellular senescence without the associated inflammatory secretory phenotype. *J. Biol. Chem.* 286 36396–36403 10.1074/jbc.M111.257071 21880712 PMC3196093

[B20] d’Adda di Fagagna F. (2008). Living on a break: cellular senescence as a DNA-damage response. *Nat. Rev. Cancer* 8 512–522 10.1038/nrc2440 18574463

[B21] d’Adda di Fagagna F. Reaper P. M. Clay-Farrace L. Fiegler H. Carr P. Von Zglinicki T. (2003). A DNA damage checkpoint response in telomere-initiated senescence. *Nature* 426 194–198 10.1038/nature02118 14608368

[B22] Demaria M. Ohtani N. Youssef S. A. Rodier F. Toussaint W. Mitchell J. R. (2014). An essential role for senescent cells in optimal wound healing through secretion of PDGF-AA. *Dev. Cell* 31 722–733 10.1016/j.devcel.2014.11.012 25499914 PMC4349629

[B23] Dickey J. S. Baird B. J. Redon C. E. Avdoshina V. Palchik G. Wu J. (2012). Susceptibility to bystander DNA damage is influenced by replication and transcriptional activity. *Nucleic Acids Res.* 40 10274–10286 10.1093/nar/gks795 22941641 PMC3488239

[B24] Dickey J. S. Baird B. J. Redon C. E. Sokolov M. V. Sedelnikova O. A. Bonner W. M. (2009). Intercellular communication of cellular stress monitored by gamma-H2AX induction. *Carcinogenesis* 30 1686–1695 10.1093/carcin/bgp192 19651821 PMC2757548

[B25] Finnberg N. Gruber J. J. Fei P. Rudolph D. Bric A. Kim S. H. (2005). DR5 knockout mice are compromised in radiation-induced apoptosis. *Mol. Cell. Biol.* 25 2000–2013 10.1128/MCB.25.5.2000-2013.2005 15713653 PMC549384

[B26] Freund A. Patil C. K. Campisi J. (2011). p38MAPK is a novel DNA damage response-independent regulator of the senescence-associated secretory phenotype. *EMBO J.* 30 1536–1548 10.1038/emboj.2011.69 21399611 PMC3102277

[B27] Gasser S. Orsulic S. Brown E. J. Raulet D. H. (2005). The DNA damage pathway regulates innate immune system ligands of the NKG2D receptor. *Nature* 436 1186–1190 10.1038/nature03884 15995699 PMC1352168

[B28] Guo Z. Kozlov S. Lavin M. F. Person M. D. Paull T. T. (2010). ATM activation by oxidative stress. *Science* 330 517–521 10.1126/science.1192912 20966255

[B29] Hagelstrom R. T. Askin K. F. Williams A. J. Ramaiah L. Desaintes C. Goodwin E. H. (2008). DNA-PKcs and ATM influence generation of ionizing radiation-induced bystander signals. *Oncogene* 27 6761–6769 10.1038/onc.2008.276 18679419

[B30] Harper J. W. Elledge S. J. (2007). The DNA damage response: ten years after. *Mol. Cell* 28 739–745 10.1016/j.molcel.2007.11.015 18082599

[B31] Havaki S. Kotsinas A. Chronopoulos E. Kletsas D. Georgakilas A. Gorgoulis V. G. (2014). The role of oxidative DNA damage in radiation induced bystander effect. *Cancer Lett.* 356 43–51 10.1016/j.canlet.201401.023 24530228

[B32] Huang T. T. Wuerzberger-Davis S. M. Wu Z. H. Miyamoto S. (2003). Sequential modification of NEMO/IKKgamma by SUMO-1 and ubiquitin mediates NF-kappaB activation by genotoxic stress. *Cell* 115 565–576 10.1016/S0092-8674(03)00895-X 14651848

[B33] Hubackova S. Krejcikova K. Bartek J. Hodny Z. (2012). IL1- and TGFbeta-Nox4 signaling, oxidative stress and DNA damage response are shared features of replicative, oncogene-induced, and drug-induced paracrine ‘bystander senescence.’ *Aging (Albany NY)* 4 932–951. 23385065 10.18632/aging.100520 PMC3615160

[B34] Iannello A. Thompson T. W. Ardolino M. Lowe S. W. Raulet D. H. (2013). p53-dependent chemokine production by senescent tumor cells supports NKG2D-dependent tumor elimination by natural killer cells. *J. Exp. Med.* 210 2057–2069 10.1084/jem.20130783 24043758 PMC3782044

[B35] Iyer R. Lehnert B. E. Svensson R. (2000). Factors underlying the cell growth-related bystander responses to alpha particles. *Cancer Res.* 60 1290–1298. 10728689

[B36] Jackson S. P. Bartek J. (2009). The DNA-damage response in human biology and disease. *Nature* 461 1071–1078 10.1038/nature08467 19847258 PMC2906700

[B37] Kang T. W. Yevsa T. Woller N. Hoenicke L. Wuestefeld T. Dauch D. (2011). Senescence surveillance of pre-malignant hepatocytes limits liver cancer development. *Nature* 479 547–551 10.1038/nature10599 22080947

[B38] Kirshner J. Jobling M. F. Pajares M. J. Ravani S. A. Glick A. B. Lavin M. J. (2006). Inhibition of transforming growth factor-beta1 signaling attenuates ataxia telangiectasia mutated activity in response to genotoxic stress. *Cancer Res.* 66 10861–10869 10.1158/0008-5472.CAN-06-2565 17090522

[B39] Klammer H. Kadhim M. Iliakis G. (2010). Evidence of an adaptive response targeting DNA nonhomologous end joining and its transmission to bystander cells. *Cancer Res.* 70 8498–8506 10.1158/0008-5472.CAN-10-1181 20861183

[B40] Ko M. Lao X. Y. Kapadia R. Elmore E. Redpath J. L. (2006). Neoplastic transformation in vitro by low doses of ionizing radiation: role of adaptive response and bystander effects. *Mutat. Res.* 597 11–17 10.1016/j.mrfmmm.2005.08.013 16414089

[B41] Krtolica A. Parrinello S. Lockett S. Desprez P. Y. Campisi J. (2001). Senescent fibroblasts promote epithelial cell growth and tumorigenesis: a link between cancer and aging. *Proc. Natl. Acad. Sci. U.S.A.* 98 12072–12077 10.1073/pnas.211053698 11593017 PMC59769

[B42] Kuilman T. Michaloglou C. Vredeveld L. C. Douma S. Van Doorn R. Desmet C. J. (2008). Oncogene-induced senescence relayed by an interleukin-dependent inflammatory network. *Cell* 133 1019–1031 10.1016/j.cell.2008.03.039 18555778

[B43] Laberge R. M. Adler D. Demaria M. Mechtouf N. Teachenor R. Cardin G. B. (2013). Mitochondrial DNA damage induces apoptosis in senescent cells. *Cell Death Dis.* 4:e727 10.1038/cddis.2013.199 PMC3730395 23868060

[B44] Lam A. R. Le Bert N. Ho S. S. Shen Y. J. Tang M. L. Xiong G. M. (2014). RAE1 ligands for the NKG2D receptor are regulated by STING-dependent DNA sensor pathways in lymphoma. *Cancer Res.* 74 2193–2203 10.1158/0008-5472.CAN-13-1703 24590060 PMC4229084

[B45] Little J. B. Azzam E. I. De Toledo S. M. Nagasawa H. (2002). Bystander effects: intercellular transmission of radiation damage signals. *Radiat. Prot. Dosimetry* 99 159–162 10.1093/oxfordjournals.rpd.a006751 12194273

[B46] Lopez-Otin C. Blasco M. A. Partridge L. Serrano M. Kroemer G. (2013). The hallmarks of aging. *Cell* 153 1194–1217 10.1016/j.cell.2013.05.039 23746838 PMC3836174

[B47] Malaquin N. Vercamer C. Bouali F. Martien S. Deruy E. Wernert N. (2013). Senescent fibroblasts enhance early skin carcinogenic events via a paracrine MMP-PAR-1 axis. *PLoS ONE* 8:e63607 10.1371/journal.pone.0063607 PMC3651095 23675494

[B48] Miyamoto S. (2011). Nuclear initiated NF-kappaB signaling: NEMO and ATM take center stage. *Cell Res.* 21 116–130 10.1038/cr.2010.179 21187855 PMC3193401

[B49] Moiseeva O. Mallette F. A. Mukhopadhyay U. K. Moores A. Ferbeyre G. (2006). DNA damage signaling and p53-dependent senescence after prolonged beta-interferon stimulation. *Mol. Biol. Cell* 17 1583–1592 10.1091/mbc.E05-09-0858 16436515 PMC1415317

[B50] Morgan W. F. (2003). Non-targeted and delayed effects of exposure to ionizing radiation: I. Radiation-induced genomic instability and bystander effects in vitro. *Radiat. Res.* 159 567–580 10.1667/0033-7587(2003)159[0567:NADEOE]2.0.CO;2 12710868

[B51] Nagasawa H. Little J. B. (1992). Induction of sister chromatid exchanges by extremely low doses of alpha-particles. *Cancer Res.* 52 6394–6396. 1423287

[B52] Nagasawa H. Little J. B. (2002). Bystander effect for chromosomal aberrations induced in wild-type and repair deficient CHO cells by low fluences of alpha particles. *Mutat. Res.* 508 121–129 10.1016/S0027-5107(02)00193-8 12379467

[B53] Nelson G. Wordsworth J. Wang C. Jurk D. Lawless C. Martin-Ruiz C. (2012). A senescent cell bystander effect: senescence-induced senescence. *Aging Cell* 11 345–349 10.1111/j.1474-9726.2012.00795.x 22321662 PMC3488292

[B54] Ohanna M. Giuliano S. Bonet C. Imbert V. Hofman V. Zangari J. (2011). Senescent cells develop a PARP-1 and nuclear factor-{kappa}B-associated secretome (PNAS). *Genes Dev.* 25 1245–1261 10.1101/gad.625811 21646373 PMC3127427

[B55] Perez-Mancera P. A. Young A. R. Narita M. (2014). Inside and out: the activities of senescence in cancer. *Nat. Rev. Cancer* 14 547–558 10.1038/nrc3773 25030953

[B56] Rodier F. Campisi J. (2011). Four faces of cellular senescence. *J. Cell Biol.* 192 547–556 10.1083/jcb.201009094 21321098 PMC3044123

[B57] Rodier F. Campisi J. Bhaumik D. (2007). Two faces of p53: aging and tumor suppression. *Nucleic Acids Res.* 35 7475–7484 10.1093/nar/gkm744 17942417 PMC2190721

[B58] Rodier F. Coppe J. P. Patil C. K. Hoeijmakers W. A. Munoz D. P. Raza S. R. (2009). Persistent DNA damage signalling triggers senescence-associated inflammatory cytokine secretion. *Nat. Cell Biol.* 11 973–979 10.1038/ncb1909 19597488 PMC2743561

[B59] Rodier F. Munoz D. P. Teachenor R. Chu V. Le O. Bhaumik D. (2011). DNA-SCARS: distinct nuclear structures that sustain damage-induced senescence growth arrest and inflammatory cytokine secretion. *J. Cell Sci.* 124 68–81 10.1242/jcs.071340 21118958 PMC3001408

[B60] Rogakou E. P. Boon C. Redon C. Bonner W. M. (1999). Megabase chromatin domains involved in DNA double-strand breaks in vivo. *J. Cell Biol.* 146 905–916 10.1083/jcb.146.5.905 10477747 PMC2169482

[B61] Rogakou E. P. Pilch D. R. Orr A. H. Ivanova V. S. Bonner W. M. (1998). DNA double-stranded breaks induce histone H2AX phosphorylation on serine 139. *J. Biol. Chem.* 273 5858–5868 10.1074/jbc.273.10.5858 9488723

[B62] Salminen A. Kauppinen A. Kaarniranta K. (2012). Emerging role of NF-kappaB signaling in the induction of senescence-associated secretory phenotype (SASP). *Cell. Signal.* 24 835–845 10.1016/j.cellsig.2011.12.006 22182507

[B63] Sedelnikova O. A. Nakamura A. Kovalchuk O. Koturbash I. Mitchell S. A. Marino S. A. (2007). DNA double-strand breaks form in bystander cells after microbeam irradiation of three-dimensional human tissue models. *Cancer Res.* 67 4295–4302 10.1158/0008-5472.CAN-06-4442 17483342

[B64] Senturk S. Mumcuoglu M. Gursoy-Yuzugullu O. Cingoz B. Akcali K. C. Ozturk M. (2010). Transforming growth factor-beta induces senescence in hepatocellular carcinoma cells and inhibits tumor growth. *Hepatology* 52 966–974 10.1002/hep.23769 20583212

[B65] Shao C. Folkard M. Prise K. M. (2008). Role of TGF-beta1 and nitric oxide in the bystander response of irradiated glioma cells. *Oncogene* 27 434–440 10.1038/sj.onc.1210653 17621264 PMC3016606

[B66] Sokolov M. V. Dickey J. S. Bonner W. M. Sedelnikova O. A. (2007). gamma-H2AX in bystander cells: not just a radiation-triggered event, a cellular response to stress mediated by intercellular communication. *Cell Cycle* 6 2210–2212 10.4161/cc.6.18.4682 17881892

[B67] Sokolov M. V. Smilenov L. B. Hall E. J. Panyutin I. G. Bonner W. M. Sedelnikova O. A. (2005). Ionizing radiation induces DNA double-strand breaks in bystander primary human fibroblasts. *Oncogene* 24 7257–7265 10.1038/sj.onc.1208886 16170376

[B68] Stilmann M. Hinz M. Arslan S. C. Zimmer A. Schreiber V. Scheidereit C. (2009). A nuclear poly(ADP-ribose)-dependent signalosome confers DNA damage-induced IkappaB kinase activation. *Mol. Cell.* 36 365–378 10.1016/j.molcel.2009.09.032 19917246

[B69] Sun Y. Campisi J. Higano C. Beer T. M. Porter P. Coleman I. (2012). Treatment-induced damage to the tumor microenvironment promotes prostate cancer therapy resistance through WNT16B. *Nat. Med.* 18 1359–1368 10.1038/nm.2890 22863786 PMC3677971

[B70] Sun Y. Nelson P. S. (2012). Molecular pathways: involving microenvironment damage responses in cancer therapy resistance. *Clin. Cancer Res.* 18 4019–4025 10.1158/1078-0432.CCR-11-0768 22619305 PMC3549396

[B71] Tanaka T. Halicka H. D. Huang X. Traganos F. Darzynkiewicz Z. (2006). Constitutive histone H2AX phosphorylation and ATM activation, the reporters of DNA damage by endogenous oxidants. *Cell Cycle* 5 1940–1945 10.4161/cc.5.17.3191 16940754 PMC3488278

[B72] Tchkonia T. Zhu Y. Van Deursen J. Campisi J. Kirkland J. L. (2013). Cellular senescence and the senescent secretory phenotype: therapeutic opportunities. *J. Clin. Invest.* 123 966–972 10.1172/JCI64098 23454759 PMC3582125

[B73] Wu C. J. Conze D. B. Li T. Srinivasula S. M. Ashwell J. D. (2006). Sensing of Lys 63-linked polyubiquitination by NEMO is a key event in NF-kappaB activation [corrected]. *Nat. Cell Biol.* 8 398–406 10.1038/ncb1384 16547522

[B74] Wu G. S. Burns T. F. Mcdonald E. R. 3rd Jiang W. Meng R. Krantz I. D. (1997). KILLER/DR5 is a DNA damage-inducible p53-regulated death receptor gene. *Nat. Genet.* 17 141–143 10.1038/ng1097-141 9326928

[B75] Xue W. Zender L. Miething C. Dickins R. A. Hernando E. Krizhanovsky V. (2007). Senescence and tumour clearance is triggered by p53 restoration in murine liver carcinomas. *Nature* 445 656–660 10.1038/nature05529 17251933 PMC4601097

